# Supplementary figures and images for: Long-term adaptation following influenza A virus host shifts results in increased within-host viral fitness due to higher replication rates, broader dissemination within the respiratory epithelium and reduced tissue damage
Source: PLoS Pathog. 2021 Dec 17;17(12):e1010174. doi: 10.1371/journal.ppat.1010174 (PMC8735595; doi:10.1371/journal.ppat.1010174)

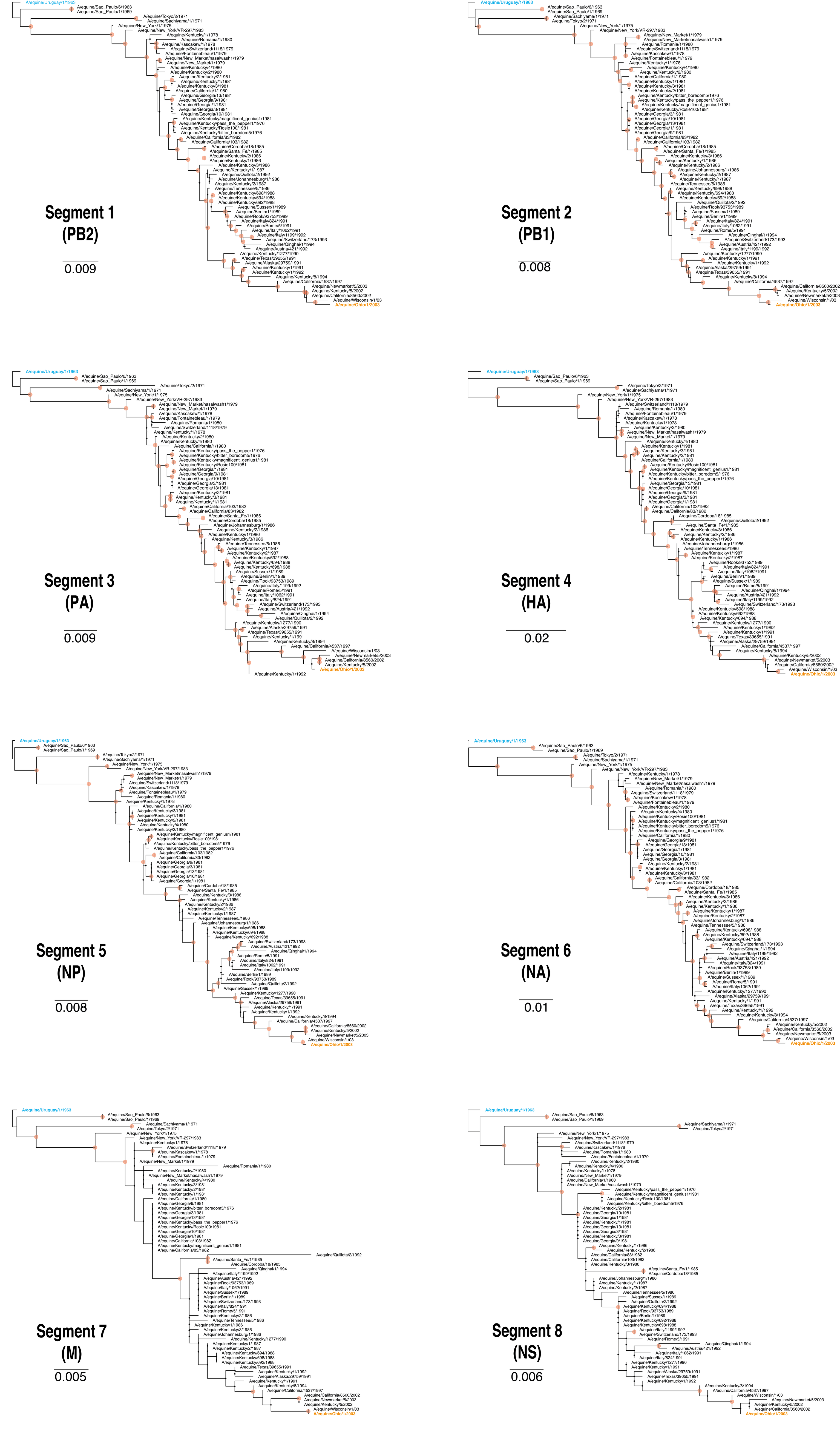

Supplement: S1 Fig — Maximum likelihood trees using 136 complete H3N8 EIV genomes. Each tree represents the phylogenetic relationship inferred for each of the eight viral genomic segments. The name of each genomic segment is indicated as follows: PB2 (polymerase basic 2); PB1 (polymerase basic 1); PA (polymerase acidic); HA (hemagglutinin); NP (nucleoprotein); NA (neuraminidase); M (matrix); and NS (non-structural). EIV/63 is indicated in cyan and EIV/2003 in orange. Nodes supported by a bootstrap value ≥ 75 are shown in red. (TIF) [file ppat.1010174.s001.tif]

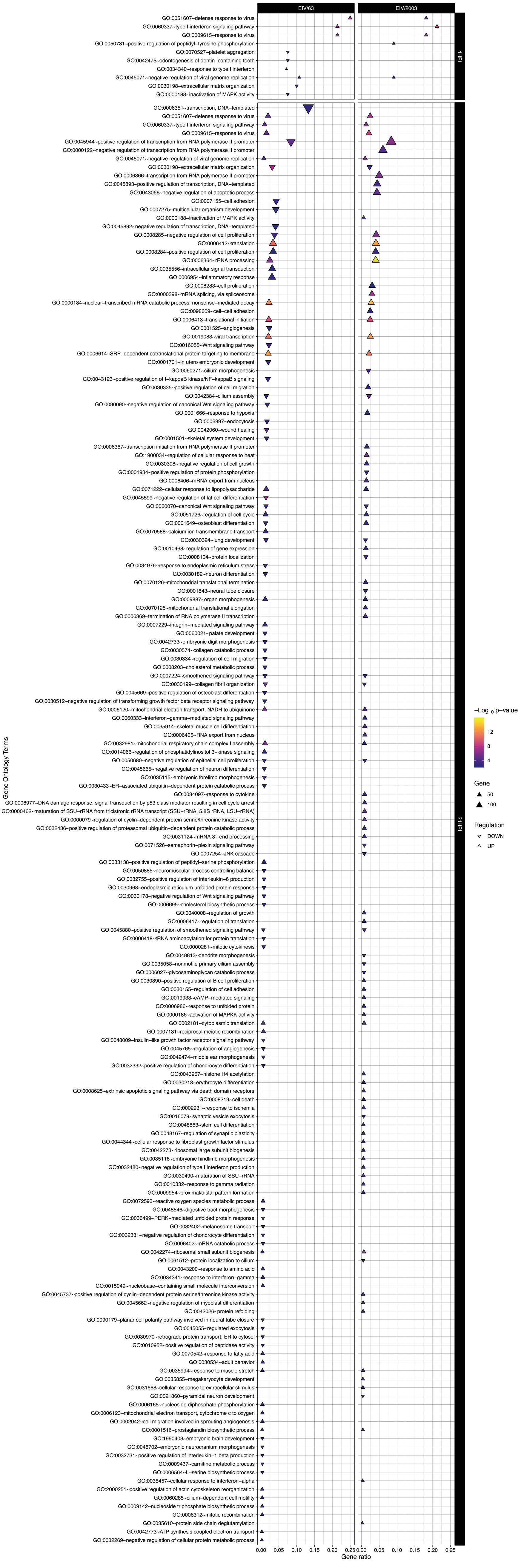

Supplement: S2 Fig — Significantly upregulated and downregulated GO terms are displayed as triangles or reverse triangles, respectively. The size of each triangle represents the number of DEGs fitting the specified GO term, while the ratio of total genes involved in each category is displayed on the x-axis. Triangles are coloured according to a blue-red-yellow gradient representing the enrichment significance (-Log10 p-value). (TIF) [file ppat.1010174.s002.tif]

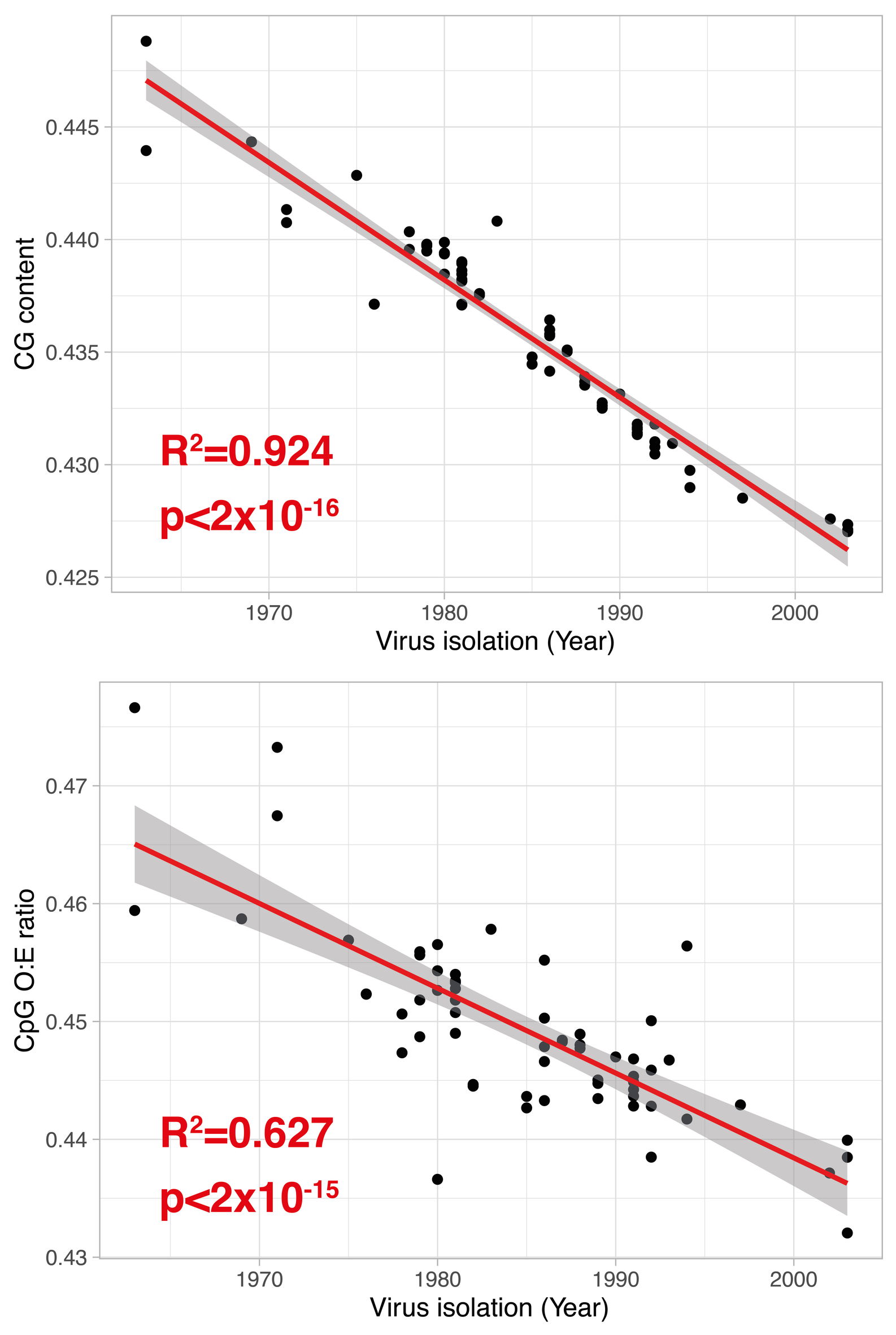

Supplement: S3 Fig — The top and bottom scatter plots show the evolution of CG content, and CpG observed/expected ratio (CpG O:E Ratio), respectively, calculated from 136 H3N8 EIV complete genomes isolated between 1963 to 2003. Linear regressions between virus isolation and either CG content or CpG O:E ratio are shown in red, while 95% CI are shown in grey. P-values and R2 are shown in red for each scatter plot. (TIF) [file ppat.1010174.s003.tif]

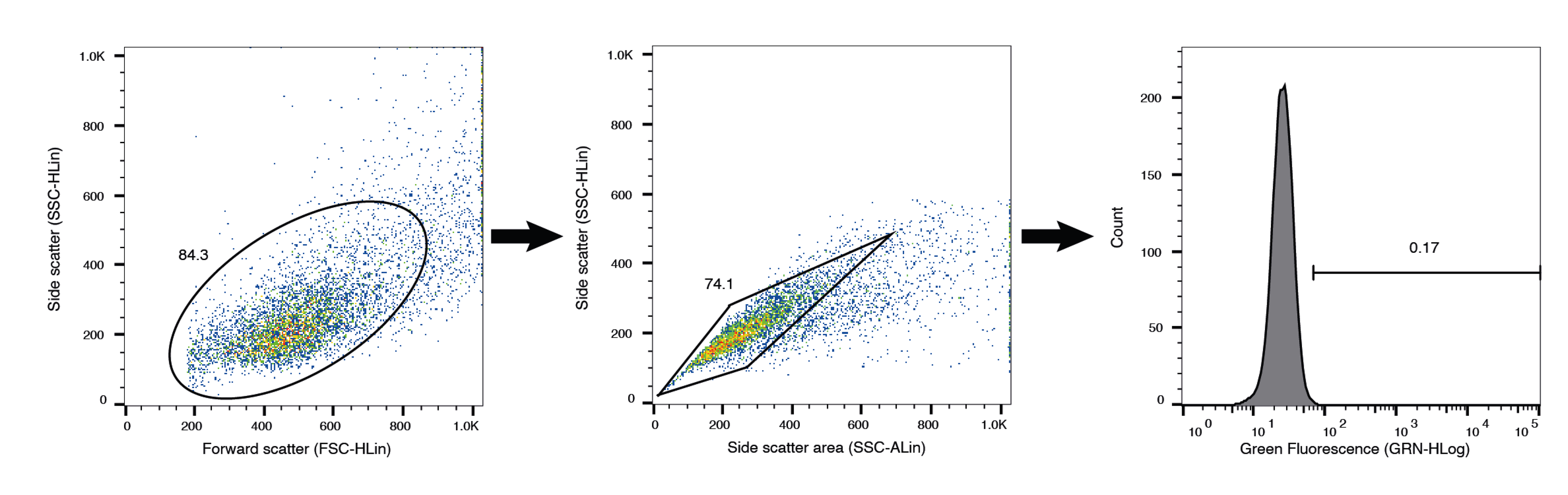

Supplement: S4 Fig — The scatter plots and density plot show from left to right the gates used to distinguish viable E.Derm (FSC-H/SSC-H), singlet (SCC-A/SSC-H), and GFP+ cells in mock-infected condition. The percentage on gated cells is given on each of the three plots. (TIF) [file ppat.1010174.s004.tif]

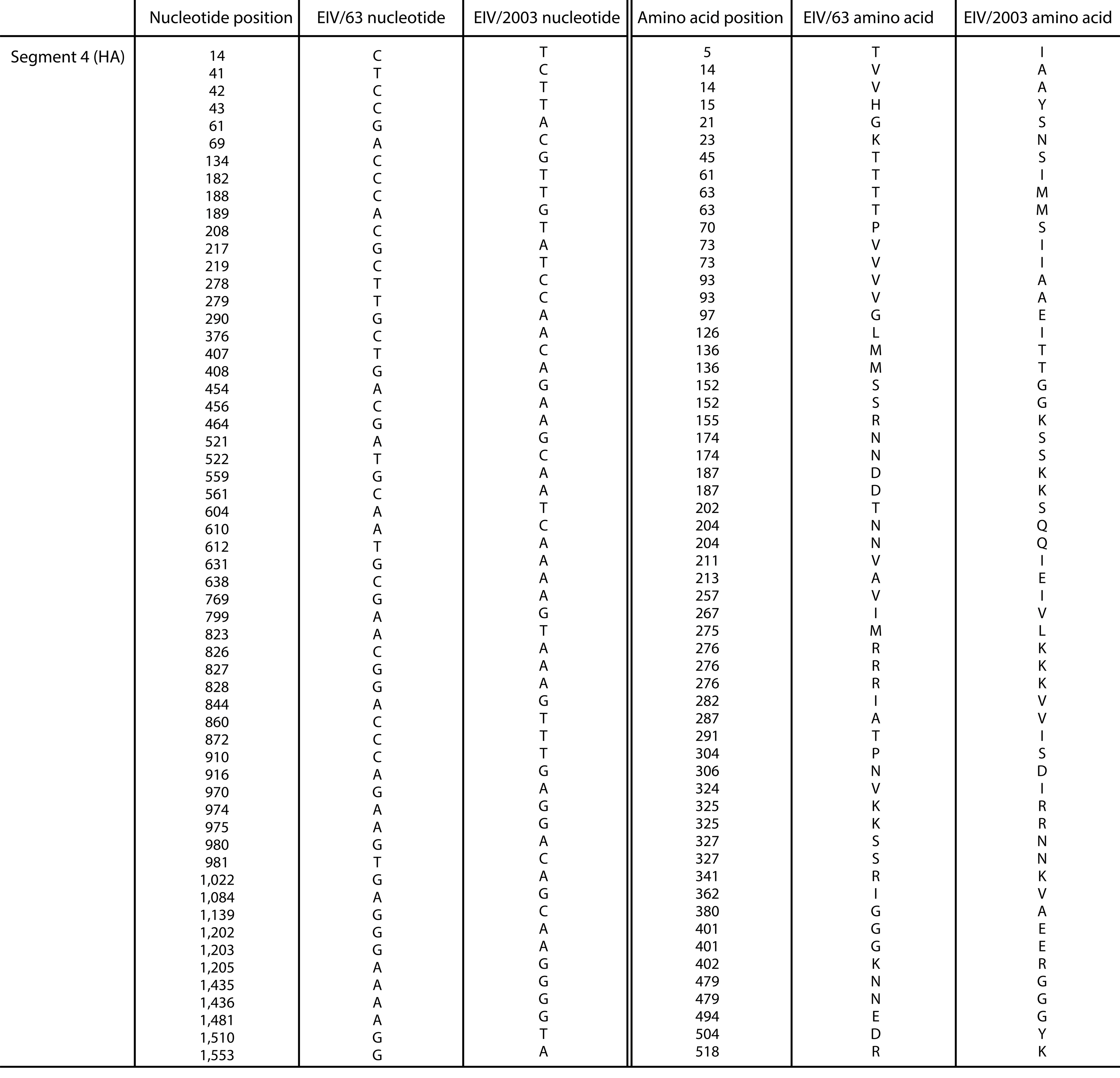

Supplement: S1 Table — Nucleotide and amino acid positions are listed. (TIF) [file ppat.1010174.s005.tif]
