# Supplementary material for: Long-term adaptation following influenza A virus host shifts results in increased within-host viral fitness due to higher replication rates, broader dissemination within the respiratory epithelium and reduced tissue damage
Source: PLoS Pathog. 2021 Dec 17;17(12):e1010174. doi: 10.1371/journal.ppat.1010174 (PMC8735595; doi:10.1371/journal.ppat.1010174)
Supplement: Attachment — Submitted filename: Rebuttal letter_submission.docx [file ppat.1010174.s008.docx]

We thank the Editor for handling this manuscript and the three reviewers for reviewing it. After reading the Editor’s and the reviewers’ comments, we agree that our manuscript lacked clarity as well as some information to put our experimental design, results, and discussion in the right context. In this modified version, we have addressed all the points raised by the reviewers. We have i) rewritten the manuscript focusing on within-host fitness rather than on virulence as the latter should consider in vivo experiments; ii) provided better background information to clarify some issues raised by the reviewers; iii) performed additional experiments and transcriptomics analyses to show how long-term virus adaptation impacts on the interferon response (the primary barrier against virus infection); and iv) have included new statistical analyses.

**Editor’s comments**

Although the reviewers find your work of interest, two of three reviewers raised some important concerns.

One referee asks for the degree of phylogenetic divergence and amino acid differences between 1963 and 2003 viruses as it is unclear from the selection criteria whether the two equine strains in this study truly represent early-introduction and contemporary virus isolates.

*The H3N8 equine influenza virus (EIV) lineage is of avian and monophyletic origin and has been circulating uninterruptedly in horses for almost 60 years. This is one of the longest periods that any avian-origin IAV has been circulating in a mammalian host in nature, therefore providing unique opportunities to study long-term adaptation. The viruses used in this study represent the earliest (A/equine/Uruguay/1963, EIV/63) and a contemporary (A/equine/Ohio/2003, EIV/2003) representative of the H3N8 equine influenza virus (EIV) lineage. We included additional text in the introduction to make this clearer (lines 96-98 and 105-111), references (15, 18-21 in the main text) and a supplementary figure showing 8 phylogenetic trees (one for each genomic segment) to make this clear (Supplementary figure 1). In addition, we produced a new panel in Figure 1 showing the number and distribution of mutations that differentiate EIV/63 and EIV/2003 (lines 142 and new panel in Figure 1 (Figure 1A).*

Two referees think there is a lack of in vivo data to support the differences in pathogenicity.

*The use of the word pathogenicity in the previous version of the manuscript was a mistake, we corrected that now. Our explant system enables us to quantify and compare differences in tissue pathogenicity, which we also referred to as tissue damage. Tissue pathogenicity/damage refers to virus-induced morphological changes in the respiratory epithelium and is one of the various fitness components we quantified to assess changes in overall virus fitness. Explants and air-liquid interface (ALI) cultures of respiratory epithelium represent to a great extent the natural site of infection of respiratory viruses and therefore are broadly used to study the infection biology of most respiratory viruses including IAVs, SARS-CoV-2 and RSV to name a few (1-3). In fact, experimental infections of pigs and ALI-cultures derived from pigs have shown an excellent correlation in infection phenotypes (4). Analyses of lesions were carried out by two Veterinary Pathologists (co-authors Francesco Marchesi and Veronica Patton) and the lesions observed are consistent with those observed in vivo in experimental infections of horses with EIV (5). The highly controlled setting of our explant infections, together with the unbiased image analyses used enabled us not only to quantify histopathological changes directly induced by the viruses, but also the biological processes that underpin them, such as apoptosis, cell division and innate immune activation. For clarity, the term* ***pathogenicity*** *has been replaced by* ***tissue pathogenicity*** *and* ***tissue damage*** *throughout the text.*

Also, concern was raised over the lack of interpretation in multidimensional scaling and transcriptomic analyses and that the study is largely descriptive, with no specific molecular or mechanistic interactions between the two viruses identified that are associated with phenotypic or host response.

*The new version of the manuscript has additional text to aid the interpretation of the MDS plots included in Figure 2 (lines 175-183). We also highlighted the transcriptome datapoints in figure 2A and 2B to help with the visualisation and interpretation of the figure.*

*With regards to the comment of the study being “largely descriptive…”. We understand that some studies aim to identify individual mutations within a virus protein, its effect on infection phenotype, and the molecular mechanism underpinning the observed difference. We have done this in previous work when we examined differences in EIV NS1(6). However, identifying individual mutations and their effect on EIV adaptation was not the aim of our study. Our study aims to assess the role of genomic evolution (not individual mutations) in virus adaptation after a host shift. We respectfully disagree with the statement that this is a largely descriptive study. This is a hypothesis-driven study (with a clear hypothesis formulated in the introduction [lines 123-125]: “We hypothesized that the accumulation of mutations across the entire H3N8 EIV genome, as a consequence of continuous evolution in horses, resulted in changes in virus-host interactions that would impact virus-induced pathology and overall fitness.”]). To test our hypothesis, we carried out a comparative study that required in-depth characterisation of the infection phenotypes of two viruses separated by 40 years of continuous evolution within a lineage of an emerging virus. Our comparative approach allowed us to identify clear differences in infection phenotypes, and to link them to differences in virus host-interactions as revealed by unbiased transcriptomics analyses. To support our results in a more relevant setting, we performed experimental infections of respiratory explants that not only showed differences in infection phenotypes within the respiratory tract of the natural host, but also the biological processes underpinning them. Finally, as the experimental design is evolutionary-guided, our data provide insight on the mechanistic processes that underpin virus adaptation.*

**Reviewers’ comments**

**Reviewer #1:**

In this study, Amat and colleagues employ two equine H3N8 influenza viruses (from 1963 and 2003, to represent early and contemporary viruses from this lineage) to study phenotypic changes between the viruses that could speak to patterns of long-term virus adaptation within a specific host. These two viruses are compared for fitness advantages in standard replication kinetics in two cell lines and ex vivo equine tracheal explants, histopathological staining in tracheal explants, and transcriptomic differences post-infection in an equine cell line (via RNAseq). ***Authors conclude that adaptation to this species over time has led to reduced viral pathogenicity.*** Studies evaluating how zoonotic viruses change and adapt to new mammalian hosts over time can provide valuable information to the literature. ***However, without in vivo data in a relevant mammalian species to support differences in pathogenicity, or without identification via mechanistic studies to ascertain the molecular determinants of the host response or virus replication changes identified via transcriptomic analyses, the results are difficult to fully interpret***.

*Our conclusion is that adaptation of EIV to its mammalian host over time has led to quantifiable changes in three key fitness components): i) virus replication; ii) within-host virus dissemination; and iii) virus-induced tissue damage. As mentioned above (and supported by references 1 to 4) ALI cultures of respiratory epithelium constitute a relevant model system to study cellular changes directly induced by the virus at its natural site of infection, and lesions observed in our ex vivo cultures are consistent with those observed in vivo in horses (5). As virus-host interactions at the site of infection are the most upstream events that occur during infection and dictate downstream biological processes within the infected host, it is reasonable to think that the observed differences in the fitness components measured will have an impact on the overall fitness of EIV.*

*Also as mentioned above our study does not aim to identify the effect of individual mutations, it aims to assess the impact of genomic evolution (i.e. mutations acquired over time across the entire genome) on viral fitness. Figure 1 shows that there are at least 833 nucleotide changes (of which 169 result in amino acid changes) between EIV/63 and EIV/2003. It is highly unlikely that an individual mutation causes the whole palette of changes described in our study because adaptation is polygenic and likely caused by the sum of individual mutations as well as epistatic effects. We added text to emphasise this point (lines 345-348). In fact, a previous study that examined canine parvovirus adaptation also showed that multiple mutations (and not single mutations) recapitulate an adaptive phenotype (lines 364-366 and new reference 45 in the main text).*

The authors do not provide sufficient background/contextual information regarding the two H3N8 viruses in this study for the authors to readily interpret results.

*We have now provided more contextual information about the H3N8 equine influenza virus lineage in the introduction (Lines 94 to 121), and in the results section (lines 141-145).*

How many amino acid differences are present between these viruses (and in what genes)? How closely are they linked phylogenetically?

*There are 169 amino acid differences between EIV/63 and EIV/2003. We added some text (lines 141-143) and a modified Figure 1 (we added panel 1A) that shows the number and distribution of synonymous and nonsynonymous mutations across the entire viral genome. We also explained more clearly the phylogenetic relationship between EIV/63 and EIV/2003 in the text (lines 143-145) and added a new supplementary Figure (Supplementary Figure 1) supporting that EIV/63 is the earliest virus of the H3N8 EIV lineage and is a monophyletic ancestor of EIV/2003. We did not include this information as new results as they had been previous published by us and others (7, 8).*

Do they exhibit differential receptor binding properties?

*H3N8 EIVs bind preferentially to α2,3 N-glycolylneuraminic acid (NeuGcα2,3Gal), which is highly abundant in the tracheal epithelium of the horse. We have added text (lines 102-104) and a reference (reference 23 in the main text) in the introduction to clarify this.*

Authors draw conclusions from data in equine trachea explants – is this the only site of virus replication in this species or could other sites within the respiratory tract be involved (e.g. are results being shown tissue type-specific or truly indicative replication in this species)?

*Equine influenza caused by H3N8 EIV is characterised by lesions predominantly in the upper and middle respiratory tract, such as rhinitis and tracheitis, where virus antigen is often detected. We have added text (lines 98-102) and a reference (reference 22 in the main text) in the introduction to clarify this point.*

The authors state that the 2003 isolate has a fitness advantage compared to the 1963 virus, “supporting that genome wide substitutions acquired during EIV evolution are adaptive” (lines 135-6) but do not state which substitutions these are.

*As mentioned above, new Figure 1A shows the number and distribution of mutations that separate EIV/63 and EIV/2003 at the genomic level.*

Without this information it is challenging to understand if the viruses employed are truly representative of early-introduction and contemporary virus isolates, or how much is due to the presence of strain-specific phenotypes.

*The phylogenetic trees that we added as Supplementary Figure 1 show that EIV/63 is the earliest virus of the H3N8 EIV lineage and that EIV/2003 is a descendant of it that represents contemporary viruses. We have added text to clarify this (lines105-111, 128-130, and 141-145).*

-Figure 1A: Do the 1963 and 2003 viruses equally infect cells? Without this information, it is difficult for the reader to understand if differences in replication kinetics shown are due to replicative differences or just different abilities to infect cells prior to replication.

*Experimental infections shown in Figure 1 were performed at the same multiplicity of infection, ensuring that the cells were infected by the same number of infectious units. In addition, new Figure 1D shows a comparison of the growth kinetics of both viruses in the presence or absence of an IFN response. The fact that EIV/63 grows at much higher levels when the IFN response is inhibited, suggests that the replicative differences are post-entry.*

-Figure 2: Authors show multidimensional scaling plots to present differing inductions of virus-specific cellular responses, but again, without additional context, this is difficult for the reader to interpret. How would these plots look with early vs contemporary viruses of a different subtype (e.g. H3N2 viruses from the 1968 pandemic vs now), or among other H3N8 isolates? Without some sort of reference provided, the reader cannot easily understand the relative import of the data presented or magnitude of the differences identified between the two H3N8 viruses.

*The MDS plots show the relative distance of the overall transcriptome data for each replicate of each condition (mock-infected, EIV/63-infected and EIV/2003-infected) at 4 and 24 hours post-infection (Figures 2A and 2B respectively). The farther away the points are, the more dissimilar their transcriptomes regardless of which condition is taken as a reference. As seen in Figure 2A, at 4 hours post-infection the data points representing cells infected with EIV/63 or EIV/2003 overlap, suggesting that early during the infection the cellular response to both viruses is very similar (albeit different from non-infected cells). However, at 24 hours post-infection the EIV/63 data points are distant from the EIV/2003 points and from the mock-infected cells. This indicates that the cells respond differently to each virus and also that the response is time-dependent. We have added some text to clarify this point (lines 175-183).*

Lines 111-13: please provide a reference that supports the benefit of E.Derm cells for studies of virus-host interactions relative to MDCKs.

*We did not mean that E.Derm cells are better suited for studies of virus host interactions that involve any virus or any influenza A virus. E. Derm cells are better suited than MDCK cells to study equine influenza viruses because they are of equine origin and EIV infects horses. Therefore, it is logical to think that the intracellular environment of E.Derm cells is more reminiscent of the horse than the intracellular environment of MDCK cells, which are of canine origin. Our results reinforce that notion as we show that a key aspect of the observed differences between EIV/63 and EIV/2003 is the equine type I IFN response (as shown in our experiments with ruxolitinib). We clarified this point on lines 140-141 ("E.Derm cells are interferon-competent (32) and thus reflect better the intracellular context of an EIV infection in its natural host than MDCK cells."). We also added some text in the Discussion (lines 360-364).*

-Figures 1C-D: The authors show replication kinetics information between the two target viruses with no additional supplemental treatment, and then in the presence of a JAK1/2 inhibitor. However, it would be beneficial for the reader for all 4 replication curves to be on the same graph, so the reader could more directly compare the relative effect of the inhibitor on the homologous, non-treated virus in addition to how kinetics differ relative to the heterologous H3N8 virus.

*We have modified the figure following this reviewer’s suggestion (see new Figure 1D).*

-Figure 3: how many replicate cultures were employed in this experiment? Authors should better disclose how many independent cultures were tested in these experiments, and provide error bars for data in figures 5E-F accordingly.

*This was an oversight from our end. Experiments described in Figure 3 have been done 3 times independently. We have added this information in the materials and methods (lines 499-500) and also in the caption of figure 3 (lines 824-825).*

*We do not understand the comment about the error bars because all the graphs in all the figures show error bars. Maybe this is an issue with the pdf format?*

-Figure 5A, C: What is the baseline (0 day) timepoint titer values for these experiments? Low NP staining data at day 1 p.i. in Figure 5C suggests that viral titers shown in Figure 5A at this timepoint could be attributable to residual virus present in the samples and not indicative of true virus replication, so a baseline reading would be informative.

*Explants were infected with a total of 200 plaque forming units (PFUs) of virus suspended in 5 uL of medium (Methods section, line 489). This infection dose is based on previous work by us and others using various influenza viruses and explants of different animal species (9-12). Figure 6 A of the revised version shows that both viruses reach titres that are several logs higher than the input virus, and such difference cannot be explained by residual virus from the infection procedure.*

-The methods section is lacking information. Please specify the limit of detection for the titration assays employed in this study (line 350). Please specify in more detail how the tracheal explants were infected and at what MOI; this information is not present in the ‘virus titrations’ section (line 340).

*The limit of detection for the titration assays is 10 plaque forming units per millilitre (PFU/ml). Details about the infectious doses for explants were provided in the Materials and Methods section named “****Experimental infections****” (lines 483-500). Regarding the use of a specific MOI, as explants have three dimensions and various cell types, it is not possible to calculate an MOI because the number of susceptible cells cannot be calculated. This is why explants were infected with a fixed amount of virus (200PFU).*

-The statistics employed do not seem sufficiently robust – the authors employ students t-test throughout, even though there are instances (e.g. graphs in Figure 6) where three groups are present; ANOVA or a similar statistical test would be more appropriate when >2 independent groups are being conducted concurrently.

*We have reanalysed all our data using a more suitable statistical approach. This resulted in improved statistical analysis in the entirety of the paper, replacing t-tests by generalised linear models (GLMs). Additionally, we've assessed the goodness of fit of the models using a dedicated tool (DHARMa package). All of the information regarding such new analysis was described in the material and methods section (lines 564-574) as well as in the figure captions. The only exception is in Figure 1C, where we used student’s t-test because we have two groups of n=100 with a normal distribution based on Shapiro test.*

**Reviewer #2:**

The authors study infections caused by early and late strains of an emerging virus (equine influenza virus, EIV). More precisely, they use existing protocols to generate virions from a 1963 strain (the earliest known) and a 2003 strain, which they then use to perform a series of experiment. First, they infect two types of cell cultures to measure relevant life-history traits. Then they perform and analyse transcriptomics profiles from infection in these cells compared to a mock control. Finally, they use horse tracheal explants to obtain an increased level of realism. They interpret their results in the context of virulence evolution and emerging infectious diseases.

Review

Overall, I was impressed by the exhaustive biological analyses performed by the authors. Indeed, they analyse a whole palette of traits, from virus load to tissue spreading. They also go beyond the mere use of transcriptomics using gene ontology to obtain functional insights. Overall, they do only have two time points (or virus strains) to perform their comparison but I think the density of tests compensates for this. My expertise with the technical aspects of the analyses performed is very limited but I do have two concerns regarding the presentation of the results and the theoretical framework. First, the state of the art could probably be improved. Second, I think the authors need to clarify essential notions they rely on (virulence, fitness) and how their experiments fit with these notions. Finally, perhaps some more details could help (e.g. the mock treatment does not seem to be defined).

*We thank this reviewer (Samuel Alizon) for the positive comments about our manuscript and the constructive criticism.*

1.1 The way the introduction is written, the reader is led to believe that such investigation of ancestral viruses has never really been performed. Most of the assays performed here are beyond my expertise but I had the feeling that similar investigations had been performed, for instance in the case of myxoma virus in Australia. There might also be some relevant experiments for Marek disease virus or other viruses.

We have included in the Introduction (lines 71-80) and Discussion (lines 364-366) additional text and references (references 8 to 11, 45) highlighting other studies on the evolution of viral fitness. We also included in the Discussion additional text comparing our findings on changes in tissue damage with previous work carried out on Newcastle Disease Virus (a RNA virus) and Myxoma Virus (a DNA virus) (lines 405-407, references 53 and 54 in the main text).

1.2 There were several instances where I thought adding references to earlier studies might be necessary. This was particularly the case for some of the methods, which are quite short and without reference (e.g. page 14). This is the case for instance for the flow cytometry analyses. However, this is also perhaps I am more familiar with the use of flow cytometry in immunology.

*We have now included a description of the flow cytometry gating strategy (Supplementary Figure 4). We added some text in the manuscript to help the reader finding this information: “The gating strategy […] is shown in S4 Fig.” (lines 509-511 of the main text).*

2) Definitions

The authors place their study in the context of virulence evolution of emerging pathogens, leaning in particular on our review (Alizon & Michalakis 2015). I think several points could be clarified.

2.1 About fitness and life cycle, the authors write that they complete the virus life cycle but there is a part that misses, which is the transmission part. Indeed, going from one virion to virions is not enough since the duration of the infectious period matters. To be clear, I do not think this affects the results here since the ancestral strain appears to be causing shorter infections. But still, the notion of fitness needs to be clarified because very often it refers to the within-host fitness (ability to colonise cells, spread in tissues, and produce virions), which may not always coincide with the between-host fitness (infect many hosts).

*When we referred to the virus cycle, we meant the virus replication cycle at the cellular level (from virus entry to virus budding, this is probably a common bias amongst virologists). We see this reviewer’s point and modified the text extensively (including the title) to highlight that our work focuses on within-host fitness.*

2.2 A similar concern is valid for virulence, which needs to be defined. In the most generic definition, it is the decrease in host fitness but here the authors only work with cells or tissues, making it impractical. Furthermore, in the formula listed in the discussion, virulence corresponds to a mortality rate, which again is tricky here. As we argue in our review, the exact definition does not really matter as long as it affects the between-host fitness (which I think is the case here). Perhaps a way out would be to refer to pathogenicity (i.e. at a cellular level) to circumvent the problem.

*We clarified the term virulence by defining virus-induced tissue pathogenicity (also referred to as tissue damage), which is essentially a measure of pathology in our explant system. We removed the text in the Discussion (regarding the formula that measures fitness) to avoid misinterpretations.*

2.3 A point that could perhaps be further discussed to help an audience less familiar with the field of virulence evolution is that of immunopathology, which I think could perhaps explain the observed evolutionary trend. In other words, try to convey the idea some part of the virulence can be associated with fitness-positive traits (e.g. virus load) and some other part with non-adaptive traits (immunopathology).

*We thank the reviewer for this suggestion as it led to additional analyses and important new insight on our work. Immunopathology is defined as a disease caused by an immune response, and generally involves the action of immune cells such as macrophages, neutrophils and/or lymphocytes. We cannot capture pathological changes due to dysregulated responses in our explant system because it lacks immune cells. However, we explored changes in the transcriptome of E.Derm associated with inflammation pathways and found really interesting differences between the viruses. This new analysis is included in lines 249-288, more specifically 270-288) and we also included new figures (panels A, B and C in Figure 5). Briefly, we found an enhanced modulation of inflammation pathway at 24hpi in EIV/63 infected cells when compared to EIV/2003. Even more interestingly we found significant upregulation and downregulation of tnfaip3 and ccl2 genes respectively, in EIV/2003 infected cells compared to EIV/63. Tnfaip3 (also known as A20) is a well know anti-inflammatory protein, while ccl2 gene product is acting on monocyte recruitment due to its chemotaxis activity. These results would suggest an enhanced inflammation in EIV/63 infected tissue, and potential immunopathology.*

3) Clarifications

3.1 I appreciated the focused writing but sometimes additional details might be necessary. For instance, unless I missed it, the authors never explain what mock infections consist of. Similarly, I am unsure all of the bioinformatics analyses can be reproduced (I would recommend that the authors publish their scripts along with the article).

*We have added the requested information about mock infections (lines 485-486). The code used in the Bioinformatics analyses has been added as a Supplementary file (Supplementary file 1).*

3.2 I am far from being an expert in statistics but I think there are some issues with the method used by the authors to analyse temporal trends in Figure 1A, C, and D. Indeed, these are time series so the points are not independent. There are several ways to account for this I think input from an expert statistician can help.

*This point has also been raised by Reviewer 1 and we addressed it above. Briefly, we have removed the temporal analysis to focus exclusively on the difference observed among EIV/63 and EIV/2003 infected explants, during the whole experiment. Such statistical analysis has been done using generalised linear models.*

l.34: add 2 cell lines?

*Added.*

l.70-72: this sentence seems a bit strong. I think there are many experiments analysing host shifts, for instance in plant viruses and arboviruses.

*This sentence has been toned down (lines 89-92). Now it reads:* *“While advances in sequencing technologies and phylogenetics have generated a vast body of knowledge on the genomic evolution of viruses and its impact on viral emergence (13) it is not entirely clear how evolution impacts on virus-host interactions, and how the latter contribute to the establishment of new endemic viruses.”*

l.78: Define fitness here? Also, virulence is not mentioned in the introduction.

*We included a definition of viral fitness and a reference (lines 71 and reference 8 in the main text). It reads: “Viral fitness -defined as the ability of a virus to generate infectious progeny in a given environment.”*

l.93-96: Is it the epistatic effects that are lacking in previous studies? I would say that the originality of the current study is to explore the biological and more mechanistic bases for host adaptation.

*Previous studies on this subject can be divided in two:*

*1) In silico analyses of viral sequences that determined phylogenetic relationships between the viruses of the lineage and the selection pressures that shaped them. Such studies did not explore the biological consequences of lineage evolution.*

*2) Experimental studies aiming to determine the effect of individual mutations on specific mechanisms. These included the antigenic effect of mutations in the viral haemagglutinin and the effect of mutations in the NS1 protein on general gene expression and the stimulation of interferon stimulated genes.*

*Previous studies did not examine epistatic effects, neither they integrated traits and approaches as we did in this manuscript.*

l.109: As discussed above, I think that part of the life-cycle is missing (epidemiology and transmission to new hosts) so a clearer definition is perhaps needed.

*We clarified this point in lines 81-89. We provided a brief description of the barriers that viruses must overcome to replicate within their hosts and put that in the context of fitness. We have also described more clearly the fitness components we measured (lines 145 to 147).*

l.133-135: Here and/or in the discussion, perhaps be more specific about why these changes are adaptative and at which level (within-host or between-host). For instance, a possibility could be to mention the duration of the infectious period.

*This has been addressed in the Discussion, which has been extensively rewritten.*

l.177-181: Can this be interpreted in terms of immune evasion of EIV/2003 and immunopathology generated by EIV/63?

*Tripartite motif (TRIM) proteins participate in multiple cellular processes. Many TRIM proteins are key players in the host antiviral defence. Some TRIM proteins directly block specific steps of the virus replication cycle while others regulate intracellular pathways induced by innate immune sensors and as a result, they modulate cytokine responses. Trim45 gene product is associated with the IFN-gamma signalling pathway, which in turn has immunoregulatory effects (*[*https://doi.org/10.3390/cells10030708*](https://doi.org/10.3390/cells10030708)*). The observed difference in trim45 transcription could be interpreted as an evasion strategy against the innate immune response. However, as explants are not vascularised we cannot evaluate immunological changes, which are usually mediated by migratory immune cells.*

l.232 and l.238: Why did you choose these proteins?

*We chose to investigate CC3, Ki67 and MX1 proteins because they are well-established markers of apoptosis, cellular division and innate immune activation, respectively, and those three processes are key to the infection phenotype as they have a direct impact on how virus spreads within a host. Apoptosis and innate immune activation are responses that aim to stop virus spread by decreasing the number of susceptible cells by either killing them (apoptosis) or making them refractory to infection (innate immune-active cells). Cellular division aims to maintain the tissue homeostasis and at the same time increases the number of susceptible cells.*

l.250: I always find the formulation "more evolved" a bit strange but it might be just me.

*That sentence has been removed.*

l.259: Actually, I think that in their seminal article Anderson & May introduce a trade-off between virulence and recovery rate (i.e. virus strains that kill their hosts more slowly are cleared more rapidly).

*As the discussion has been almost entirely rewritten to focus more clearly on within-host fitness than virulence, this sentence has been removed.*

l.262-263: This is where the definitions are particularly important because Leggett et al. do not mention fitness. Do you mean transmission rate?

*The reviewer is right, we did not use the correct definition. As the discussion has been almost entirely rewritten to focus more clearly on within-host fitness than virulence, this sentence has been removed.*

l.279: "multiple different measures" seems a bit heavy in terms of style.

*This text has been removed.*

l.280-284: I agree but the relationship can be unclear. For instance, Legett et al find that slower growth leads to more virulence. I (and others) have also investigated cases where more rapid growth leads to faster clearance from the immune response.

*We agree.*

l.297: Again, it is important to be very clear about what virulence is and how it is measured. This is also where I would discuss the hypothesis that immunopathology might have decreased?

*Please see lines 384-407 in the Discussion, where we added clear definitions as well as references and discussed extensively the role of virulence on virus adaptation in the context of this work and the work of others.*

l.312-315: What about recovery rates? This also seems to go in the same direction. Furthermore, how lethal is EIV to horses? In other words, can virulence/mortality be a constraint per se?

*The paragraph this reviewer is alluding to has been removed. But to answer this reviewer's question, equine influenza is rarely fatal.*

l.333-339: This is a naive question but is it possible to control the number of segments per virion?

*Not that we are aware of. However, it is known that within a virus population the number of segments per virion is not always 8.*

l.390: hyphen between Benjamini and Hochberg?

*This typo has been corrected.*

**Reviewer #3:**

Amat and colleagues describes that long-term adaptation following virus host shifts induces efficient replication rate, broader intra-host spread and lower pathogenicity. The potential mechanisms and outcomes of long-term virus adaptation remain not completely understood. The authors used an early H3N8 and its descendant--- well-adapted H3N2 equine influenza viruses to infect cells and equine tracheal explants to determine their phenotypic changes. Results show that the adapted H3N8 virus showed more efficient replication, formed more big size of plaques and lower susceptibility to interferon. RNAseq and transcriptomic analysis showed host responses to each virus, major difference in influencing host immunity. Further infection study of equine respiratory explants showed the adapted virus replicated significant higher titers and infected much more cells and larger areas of the respiratory epithelium in contrast the early virus. The authors conclude that the long-term adaptation of equine influenza virus in the host results in efficient replication rate, broader intra-host spread and lower pathogenicity. This study provides insight on equine influenza virus-host interaction after long term adaption and virus fitness. There are several concerns.

1) The adapted virus showed more effective to counteract host innate immunity, especially type I IFN response based on results of virus replication in cells and RNAseq and transcriptomic analysis. There is lack of solid data from the virus aspect to further confirm it. For example, which gene (s), protein(s) or the whole virus are responsible for the phenomena.

*To confirm this result we performed additional experiments. We treated E.Derm cells with universal IFN and determined their transcriptome. This enabled us to determine the genes that are differentially expressed in a "full blown" type-I IFN response of equine cells. We then compared the level of expression of this differentially expressed IFN stimulated genes (DE-ISGs) in cells infected with EIV/63 and EIV/2003. We show that the level of transcription of DE-ISGs varies with each virus (Figure 4 in the revised version of the manuscript), providing a mechanistic explanation to the observed differences in the way that EIV/63 and EIV/2003 overcome the IFN response.*

*As mentioned above, our study aimed to assess the impact of whole genome changes across the entire genome on within-host viral fitness. As fitness is polygenic, different gene combinations are required to explain which genes are involved in the observed differences. Since IAVs possess 8 genomic segments that encode for at least 11 genes, identifying the genes that explain the observed differences is a massive combinatorial undertaking that is beyond the scope of this work.*

2)	Authors employed the equine respiratory explants in this study and concluded the adapted virus reduces pathogenicity. This point is questionable as the authors did not perform horse or pony studies infecting with both viruses in parallel. Not sure whether the results of horse infection study will be consistent with those of equine respiratory explants.

*We agree with this reviewer and modified the manuscript accordingly. We focused on within-host tissue damage. In addition, we provide additional text (lines 370-377 of the revised manuscript) and a reference supporting that the infection phenotype observed in explants is predictive of the infection phenotype in vivo (reference 49). Finally, a previous study done in horses with two H3N8 EIVs of the same lineage studied here showed that the more evolved virus was less pathogenic. As this is an important point supporting our results, we added some text and a reference in the discussion (lines 398-404 and reference 52 of the revised version of the manuscript).*

3)	The statement in the page 8 lines 221-222 “ the lower number of cells and the lower the epithelial area the more pathogenicity” is confused. Not sure the criteria are accurate or proper?

*We agree that this was confusing and removed that sentence.*

4)	EIV/63 in cells with the presence of ruxotilinib grew to one log lower than in cells in the absence of ruxotilinib in the Figure 1 C and D. The author described “ levels of virus replication remained mainly unaltered for EIV/2003 in the presence of ruxotilinib”, please confirm the result and statement accurate.

*The differences in the kinetics of viral growth for EIV/63 in the presence or absence of ruxolitinib are statistically different (Figure D, lines 162-164. Please note that we have reanalysed our data using General Linear Models and the p value=0.039). For EIV/2003, there is no statistical difference on the growth kinetics in the presence or absence of ruxolitinib (p value=0.331). We confirmed this result and also made it more clear (lines 164-166).*

Lack of detailed information in some analysis such as multidimensional scaling plots and gene ontology, not easy to understand.

*We have included additional text to make these analyses clearer. (See lines 175-183) and modified figure 2 A and B by grouping the datapoints for each replicate of each virus, to facilitate visualisation.*

References

1.	Hui KPY, Cheung MC, Perera R, Ng KC, Bui CHT, Ho JCW, et al. Tropism, replication competence, and innate immune responses of the coronavirus SARS-CoV-2 in human respiratory tract and conjunctiva: an analysis in ex-vivo and in-vitro cultures. Lancet Respir Med. 2020;8(7):687-95.

2.	Villenave R, Shields MD, Power UF. Respiratory syncytial virus interaction with human airway epithelium. Trends Microbiol. 2013;21(5):238-44.

3.	Zhou J, Wang D, Gao R, Zhao B, Song J, Qi X, et al. Biological features of novel avian influenza A (H7N9) virus. Nature. 2013;499(7459):500-3.

4.	Fu Y, Dürrwald R, Meng F, Tong J, Wu N-H, Su A, et al. Infection Studies in Pigs and Porcine Airway Epithelial Cells Reveal an Evolution of A(H1N1)pdm09 Influenza A Viruses Toward Lower Virulence. The Journal of Infectious Diseases. 2019;219(10):1596-604.

5.	Muranaka M, Yamanaka T, Katayama Y, Niwa H, Oku K, Matsumura T, et al. Time-related Pathological Changes in Horses Experimentally Inoculated with Equine Influenza A Virus. J Equine Sci. 2012;23(2):17-26.

6.	Chauche C, Nogales A, Zhu H, Goldfarb D, Ahmad Shanizza AI, Gu Q, et al. Mammalian Adaptation of an Avian Influenza A Virus Involves Stepwise Changes in NS1. J Virol. 2018;92(5).

7.	Murcia PR, Wood JL, Holmes EC. Genome-scale evolution and phylodynamics of equine H3N8 influenza A virus. J Virol. 2011;85(11):5312-22.

8.	Mino S, Mojsiejczuk L, Guo W, Zhang H, Qi T, Du C, et al. Equine Influenza Virus in Asia: Phylogeographic Pattern and Molecular Features Reveal Circulation of an Autochthonous Lineage. J Virol. 2019;93(13).

9.	Gonzalez G, Marshall JF, Morrell J, Robb D, McCauley JW, Perez DR, et al. Infection and pathogenesis of canine, equine, and human influenza viruses in canine tracheas. J Virol. 2014;88(16):9208-19.

10.	Patrono LV, Bonfante F, Zanardello C, Terregino C, Capua I, Murcia PR. Phylogenetically distinct equine influenza viruses show different tropism for the swine respiratory tract. J Gen Virol. 2015;96(Pt 5):969-74.

11.	Feng KH, Gonzalez G, Deng L, Yu H, Tse VL, Huang L, et al. Equine and Canine Influenza H3N8 Viruses Show Minimal Biological Differences Despite Phylogenetic Divergence. J Virol. 2015;89(13):6860-73.

12.	Zhu H, Damdinjav B, Gonzalez G, Patrono LV, Ramirez-Mendoza H, Amat JAR, et al. Absence of adaptive evolution is the main barrier against influenza emergence in horses in Asia despite frequent virus interspecies transmission from wild birds. PLoS Pathog. 2019;15(2):e1007531.
