# Supplementary material for: Long-term adaptation following influenza A virus host shifts results in increased within-host viral fitness due to higher replication rates, broader dissemination within the respiratory epithelium and reduced tissue damage
Source: PLoS Pathog. 2021 Dec 17;17(12):e1010174. doi: 10.1371/journal.ppat.1010174 (PMC8735595; doi:10.1371/journal.ppat.1010174)
Supplement: Attachment — Submitted filename: Rebuttal letter_submissionV2.docx [file ppat.1010174.s009.docx]

We thank the Editor and the reviewers for the very positive feedback on our manuscript. We have addressed all the points raised by the reviewers.

**Reviewers’ comments**

**Reviewer #1:**

Inclusion of a new Figure 1A is helpful to understand the magnitude of amino acid differences present between the two viruses under study, but is still lacking specificity. Considering the high number of nonsynonymous changes in the HA between the two viruses (including in HA1, which could impact receptor binding specificity), it would be beneficial if the authors provided a supplemental table to disclose exactly which amino acid changes are present in this protein between the two viruses. In PMID 21430049 (which shares authors with this study), several sites in the HA, including those linked with antigenic sites, were identified between H3N8 equine viruses over time; providing a list of amino acid differences in the HA for the two viruses under investigation here would thus be very helpful to the reader.

We have added a new Supplementary Table (Supplementary table 1), providing all the amino acids mutations in HA. We added some text to guide the reader to this new table (lines 143-146).

Lines 52 and 135: authors state they are examining differences in “infection phenotype” but are employing replication-based assays in Figure 1B and 1D to assess this; wording should be changed to replication so not to mislead the reader, and to better match how the authors describe these investigations on line 168 (“virus growth kinetics and cell to cell spread”).

The term "infection phenotype" was changed to "replication kinetics" (Line 52) and to "virus replication" (line 135).

Lines 451-454: the authors state that the plasmids for EIV/63 are derived from reference 63, and the plasmids for EIV/2003 are derived from reference 64; however, it appears that reference 63 is incomplete (is this a dissertation? Book chapter?) so the reader cannot follow what the authors are conveying, and reference 64 only mentions plasmids from EIV/63, not EIV/2003. Please ensure both of these references are correct and accurately link to the information intended.

We thank the reviewer for spotting this mistake. We have referenced the plasmids to reference 63, which is a PhD thesis. In addition, we added the link to the thesis.

The authors state in their response to reviewer comments that the limit of detection for plaque assays was 10 PFU, but did not include this information in the methods section; please add this. In this vein, Figures 1B and 1D show mock-infected cells had titers of 0 PFU/ml which would be below the limit of detection for this assay, and should be adjusted accordingly.

We added this information in the methods section (lines 475-477) and also modified the caption of Figure 1 to highlight this. Additionally, we added a dash line to the graphs on Figure 1 to show the limit of detection.
